# Vaccination coverage of primary care providers against seasonal influenza, tetanus, pneumococcal pneumonia and herpes zoster: A cross-sectional study in Greece

**DOI:** 10.3934/publichealth.2023061

**Published:** 2023-11-24

**Authors:** Panagiota Kalatzi, Antonios K. Travlos, Nickos Geladas, Maria Iliadou, Chara Tzavara, Costas Chryssanthopoulos, Alexandros Mihopoulos, Styliani Tziaferi

**Affiliations:** 1 Laboratory of Integrated Health Care, Department of Nursing, University of Peloponnese, Tripoli, Greece; 2 Department of Sports Organization and Management, University of Peloponnese, Sparti, Greece; 3 Department of Physical Education & Sport Science, National & Kapodistrian University of Athens, Athens, Greece; 4 Department of Midwifery, University of West Attica, Athens, Greece; 5 Medical School, National and Kapodistrian University of Athens, Athens, Greece

**Keywords:** vaccination, primary care providers, influenza, tetanus, herpes zoster, pneumococcal pneumonia

## Abstract

**Introduction:**

Primary care providers' (PCPs) compliance to self-immunization is important for their protection and the protection of their colleagues and patients and has been associated with the coverage of the general public. In this study, we aim to investigate the vaccination coverage of PCPs.

**Methods:**

A questionnaire-based cross-sectional survey was conducted among physicians, nurses and pharmacists employed in public or private primary care settings in Greece. Demographic and occupational characteristics as well as vaccination coverage data for influenza, tetanus, pneumococcal pneumonia and herpes zoster were collected. Statistical significance was set at 0.05.

**Results:**

In total, 748 (61.7% response rate) PCPs participated. Vaccination rates were 66.4% (496/747) for influenza (2019/2020 flu season), 62.9% (469/746) for tetanus (10-year Td or Tdap booster dose), 70% (14/20) for pneumococcal pneumonia (≥ 1 dose of PPSV23 or PCV13) and 12.3% (10/81) for herpes zoster. Multiple logistic regression revealed that nurses had significantly lower probability of being vaccinated against influenza [odds ratio (*OR*) = 0.25; 95% confidence interval (*CI*) = 0.14–0.45] and pharmacists had significantly lower probability of being vaccinated against both influenza (*OR* = 0.44; 95% *CI* = 0.31–0.62) and influenza & tetanus (*OR* = 0.52; 95% *CI* = 0.37–0.73) compared to physicians. Older age (>40 years) was an independent risk factor for not receiving a tetanus vaccine (40–49 *vs*. 19–39; *OR* = 0.42; 95% *CI* = 0.28–0.63, over 50 years old *vs*. 19–39; *OR* = 0.54; 95% *CI* = 0.36–0.79).

**Conclusions:**

The results revealed suboptimal vaccination rates among health providers who are in the frontline of adult immunization. Individualized and targeted measures to improve their vaccination coverage and indirectly the vaccination coverage of their patients, are therefore required.

## 1. Introduction

Healthcare professionals (HCPs) are at increased risk for occupational exposure to vaccine-preventable diseases. High vaccination coverage among HCPs increases their own, their colleagues' and their patients' protection and has been associated with the coverage of the general public [1],[2].

In Greece, the National Adult Immunization Schedule (NAIS) describes the vaccinations for adults, which are recommended based on age or other indication (e.g. certain health conditions, healthcare profession, etc). Specifically, policies for the immunization against influenza, hepatitis B, measles-mumps-rubella and recently the Coronavirus Disease 2019 (Covid-19) exist for all HCPs, while indications for vaccination against varicella (Chickenpox) and meningococcus exist only for microbiology personnel and for HCPs in contact with high-risk patients, respectively. In addition, besides the occupational recommended vaccines, HCPs should be up to date with routinely recommended vaccines scheduled in the NAIS, appropriate for their age or health condition [i.e. Td/ Tdap (tetanus-diphtheria / tetanus-diphtheria-pertussis), HZV (Herpes Zoster), PPSV23/ PCV13 (pneumococcal polysaccharide/ pneumococcal conjugate), HepA (Hepatitis A), Hib (Haemophilus influenzae type b) and HPV (Human Papillomavirus)] [3]. Mandatory policies are implemented only for the Covid-19 vaccination of HCPs, while all the vaccines listed on the Greek NAIS, including both the primary series and recommended booster shots, are offered free of charge to all residents in the country, whether insured or not, including asylum-seekers [4].

Vaccination services in Greece are mostly delivered in primary care settings [5]. Primary care providers (PCPs) are the driving force in maintaining and increasing vaccine uptake as they are the most involved in the promotion and delivery of vaccines and serve as role models for the general population [5]–[7]. Indeed, their immunization is very important considering its positive association with their willingness to recommend the vaccination to their patients [8]–[10]. Although the vital role of PCPs has been demonstrated in literature, the vaccination rates of PCPs have been largely unmeasured in Greece. A substantial number of published studies have assessed the vaccination coverage of HCPs employed in acute care hospitals while, to our knowledge, only a few have reported the vaccination coverage rates among PCPs during the last decade [11]–[14]. However, these surveys are limited to a particular geographical area [11],[12] or are focused only on occupational vaccines for HCPs [i.e., HepB (Hepatitis B), MMR (Measles-Mumps-Rubella), VAR (Varicella) Covid-19 vaccines] and especially on influenza [13],[14].

In light of the aforementioned information, the determination of PCPs' compliance to self-immunization may have important public health implications. Thus, in this study, we assessed PCPs' vaccination coverage against the common infection of seasonal influenza, and the routine vaccinations against tetanus, pneumococcal pneumonia and herpes zoster.

## 2. Materials and methods

### 2.1. Study design

A cross-sectional survey was conducted from September to November 2020 and enrolled a convenience sample of PCPs who had applied for participation in three national primary care scientific conferences, held annually in Greece. All registered attendees were considered potentially eligible and were invited to participate through their registrated email.

### 2.2. Participants

Our eligibility criteria for participation in the survey required that subjects list a profession as primary care physician, nurse working in primary health care and pharmacist working in primary health care or in the community and have an active employment in either the public or private sector. PCPs residing outside Greece (i.e., Cyprus, etc.) and students were excluded.

### 2.3. Questionnaire

An anonymous questionnaire was developed and distributed electronically (Google Forms software). Prior to its distribution, the questionnaire underwent preliminary pilot testing and validity evaluation by the researchers. Data were collected on PCPs demographic and occupational characteristics, and self-reported vaccination status for vaccines routinely recommended, as part of the Greek NAIS, for all adults aged ≥19 years [Td/ Tdap vaccines), for all HCPs (influenza vaccine) or indicated depending on age (pneumococcal and herpes zoster vaccination) [3]. Accordingly, a history of completed up-to-date vaccination was defined as 1 shot for seasonal influenza during the last influenza season (October 2019 to May 2020), 1 booster dose of any tetanus-toxoid containing vaccine within the last 10 years, ≥1 shot of PPSV23 or PCV13 among participants aged 65 or older and 1 shot for herpes zoster among participants aged ≥60 years. Questionnaires with more than four missing answers were excluded.

### 2.4. Ethical approval

Ethical approval was obtained from the Ethics Committee of the University of Peloponnese (No 1908/05–02–2019). Information about the study and the rights of research participants was provided in the introductory part of the e-questionnaire. All participants provided their informed consent by clicking on the appropriate button before entering the survey.

### 2.5. Statistical analysis

Qualitative variables were expressed as absolute and relative frequencies. Pearson chi-square and Fisher's exact tests were used for the comparison of proportions. Bonferroni correction was used in order to control for type I error in multiple comparisons. Logistic regression analysis was used in order to find independent factors associated with participants' vaccination. Adjusted odds ratios (OR) with 95% confidence intervals (95% *CI*) were computed from the results of the logistic regression analyses. All *p*-values reported are two-tailed. Statistical significance was set at 0.05 and analyses were performed using SPSS statistical software (version 24.0).

## 3. Results

In total, 864 potentially eligible subjects responded to the survey. The response rate was 61.7% (864/1401). Of those responded, 748 were confirmed as eligible, thus, this was the number of PCPs included in the study analysis. Among the responders, 19/748 (2.5%) did not report their sex, 7/748 (0.9%) their age, 2/748 (0.3%) their vaccination status against tetanus, 1/82 (1.2%) their vaccination status against herpes zoster and 1/748 (0.1%) against influenza. Participants who did not report their age were included only in influenza and tetanus analyses. Ages ranged between 25 and 67. Table 1 shows in detail the demographic characteristics of the surveyed population. Data about vaccination rates are presented in Table 2. Vaccination coverage rates were suboptimal for influenza, tetanus or their combination (66.4%, 62.9% and 45.6%, respectively). Only 12.3% (10/81) of PCPs aged ≥60 years had been vaccinated against herpes zoster, and only 10.0% (2/20) of PCPs aged ≥65 years had been vaccinated against seasonal influenza, tetanus, pneumococcal pneumonia and herpes zoster. The proportion of PCPs who were vaccinated for influenza was greater for physicians as compared to nurses and pharmacists (*p* < 0.001). Moreover, the vaccination rate for both influenza and tetanus vaccines was higher among physicians than in pharmacists (*p* = 0.001) (Table 3). Significant differences between sexes were found for influenza or influenza and tetanus vaccination rates, with men being vaccinated at a higher rate compared to women (Table 4). Regarding the association between age and vaccination coverage, we found a significant relationship only for tetanus where PCPs aged 19–39 had higher vaccination rates than those being 40–49 (*p* < 0.001) or older (*p* = 0.002) (Table 5). Multiple logistic regression revealed that profession and age had a significant impact on vaccination uptake (Table 6). Specifically, nurses had by 75% lower probability of being vaccinated against influenza compared to physicians (*OR* = 0.25; 95% *CI* = 0.14–0.45). Similarly, pharmacists had by 56% lower probability of being vaccinated against influenza (*OR* = 0.44; 95% *CI* = 0.31–0.62) and by 48% lower probability of being vaccinated against influenza & tetanus (*OR* = 0.52; 95% *CI* = 0.37–0.73), compared to physicians. Older age (>40 years) was an independent risk factor for not receiving a tetanus vaccine (40–49 *vs*. 19–39; *OR* = 0.42; 95% *CI* = 0.28–0.63, over 50 years old *vs*. 19–39; *OR* = 0.54; 95% *CI* = 0.36–0.79).

**Table 1.** Demographic characteristics of participants.

Characteristics	*n* (%)
Sex (*N* = 729)
Male	309 (42.4)
Female	420 (57.6)
Age group (years) (*N* = 741)
19–39	284 (38.3)
40–49	217 (29.3)
50–59	158 (21.3)
60–64	62 (8.4)
≥65	20 (2.7)
Region of residence/work (NUTS1) (*N* = 748)
Attica (EL3)	400 (53.5)
Aegean islands, Crete (EL4)	91 (12.2)
Northern Greece (EL5)	73 (9.7)
Central Greece (EL6)	184 (24.6)
Profession (*N* = 748)
Physician	453 (60.6)
Pharmacist	238 (31.8)
Nurse	57 (7.6)

Note: *N* = the total number of participants who answered per question.

**Table 2.** PCPs' vaccination rates.

Vaccines	Vaccinated *n/N* (%)
Influenza	496/747 (66.4)
Td/Tdap	469/746 (62.9)
PCV13/PPSV23*	14/20 (70.0)
Zoster**	10/81 (12.3)
Influenza and Td/Tdap	340/746 (45.6)
Influenza, Td/Tdap and Zoster**	8/81 (9.9)
Influenza, Td/Tdap, Zoster and PCV13/PPSV23*	2/20 (10.0)

Notes: *n/N* = the number of vaccinated participants/ the total number of participants who answered per question; *applies to participants aged 65 years or older; **applies to participants aged 60 years or older.

**Table 3.** PCPs' vaccination rates by profession.

Vaccines	Profession			*p*-value
	Nurse	Pharmacist	Physician	
	*n*/*N* (%)	*n*/*N* (%)	*n*/*N* (%)	
Influenza	24/57 (42.1)	135/237 (57.0)	337/453 (74.4)	<0.001^a^
Td/Tdap	42/57 (73.7)	140/237 (59.0)	287/452 (63.5)	0.135^a^
PCV13/PPSV23*	0/0 (0.0)	7/11 (63.6)	7/9 (77.8)	0.642^b^
Zoster**	0/0 (0.0)	4/41 (9.8)	6/40 (15.0)	0.519^b^
Influenza and Td/Tdap	23/57 (40.4)	89/237 (37.6)	228/452 (50.4)	0.004^a^
Influenza, Td/Tdap and Zoster**	0/0 (0.0)	3/41 (7.3)	5/40 (12.5)	0.482^b^
Influenza, Td/Tdap, Zoster and PCV13/PPSV23*	0/0 (0)	1/11 (9.1)	1/9 (11.1)	1.000^b^

Notes: *n/N* = the number of vaccinated participants/ the total number of participants who answered per question; *applies to participants aged 65 years or older; **applies to participants aged 60 years or older; ^a^Pearson's chi square test; ^b^Fisher's exact test.

**Table 4.** PCPs' vaccination rates by sex.

Vaccines	Sex		*p* Pearson's *χ^2^* test
	Male *n/N* (%)	Female *n/N* (%)	
Influenza	218/309 (70.6)	265/419 (63.2)	0.039
Td/Tdap	205/304 (67.4)	252/415 (60.7)	0.065
PCV13/PPSV23*	12/16 (75.0)	2/3 (66.7)	1.000
Zoster**	8/48 (16.7)	2/30 (6.7)	0.301
Influenza and Td/Tdap	154/308 (50.0)	178/419 (42.5)	0.044
Influenza, Td/Tdap and Zoster**	6/48(12.5)	2/30 (6.7)	0.704
Influenza, Td/Tdap, Zoster and PCV13/PPSV23*	2/16 (12.5)	0/3 (0.0)	1.000

Notes: *n/N* = the number of vaccinated participants/ the total number of participants who answered per question; *applies to participants aged 65 years or older; **applies to all participants aged 60 years or older.

**Table 5.** PCPs' vaccination rates by age group.

Vaccines	Age group			*p* Pearson's χ^2^ test
	19–39	40–49	≥50	
	*n/N* (%)	*n/N* (%)	*n/N* (%)	
Influenza	175/284 (61.6)	149/217 (68.7)	168/239 (70.3)	0.081
Td/Tdap	201/278 (72.3)	119/216 (55.1)	142/237 (59.9)	<0.001
PCV13/PPSV23*	0/0 (0.0)	0/0 (0.0)	14/20 (70.0)	-
Zoster**	0/0 (0.0)	0/0 (0.0)	10/81 (12.3)	-
Influenza and Td/Tdap	142/284 (50.0)	90/216 (41.7)	104/239 (43.5)	0.137
Influenza, Td/Tdap and Zoster**	0/0 (0.0)	0/0 (0.0)	8/81 (9.9)	-
Influenza, Td/Tdap, Zoster and PCV13/PPSV23*	0/0 (0.0)	0/0 (0.0)	2/20 (10.0)	-

Notes: *n/N* = the number of vaccinated participants/ the total number of participants who answered per question; *applies to participants aged 65 years or older; **applies to participants aged 60 years or older.

**Table 6.** Multivariate logistic regression results.

Project		*OR* (95% *CI*)‡	*p*
Influenza	Sex: Female *vs*. Male	0.83 (0.59–1.15)	0.263
	Age: 40–49 *vs*. 19–39	1.09 (0.73–1.63)	0.686
	50+ *vs*. 19–39	1.26 (0.86–1.86)	0.236
	Region of residence/work: Attica *vs*. Other	1.20 (0.86–1.65)	0.280
	Profession: Nurse *vs*. physician	0.25 (0.14–0.45)	<0.001
	Pharmacist *vs*. physician	0.44 (0.31–0.62)	<0.001
Td/Tdap	Sex: Female *vs*. Male	0.74 (0.54–1.02)	0.064
	Age: 40–49 *vs*. 19–39	0.42 (0.28–0.63)	<0.001
	50+ *vs*. 19–39	0.54 (0.36–0.79)	0.001
	Region of residence/work: Attica *vs*. Other	1.05 (0.76–1.44)	0.775
	Profession: Nurse *vs*. physician	1.68 (0.88–3.20)	0.113
	Pharmacist *vs*. physician	0.85 (0.61–1.20)	0.360
PCV13/ PPSV23*^a^	Sex: Female *vs*. Male	0.67 (0.02–18.06)	0.810
	Region of residence/work: Attica *vs*. Other	0.01 (0.00–0.01)	0.999
	Profession: Pharmacist *vs*. physician	0.75 (0.06–8.83)	0.819
Zoster**^a^	Sex: Female *vs*. Male	0.41 (0.08–2.14)	0.288
	Region of residence/work: Attica *vs*. Other	0.35 (0.08–1.51)	0.159
	Profession: Pharmacist *vs*. physician	0.69 (0.17–2.82)	0.609
Influenza and Td/Tdap	Sex: Female *vs*. Male	0.75 (0.55–1.01)	0.057
	Age: 40–49 *vs*. 19–39	0.90 (0.67–1.21)	0.490
	50+ *vs*. 19–39	0.75 (0.53–1.07)	0.112
	Region of residence/work: Attica *vs*. Other	0.99 (0.73–1.34)	0.957
	Profession: Nurse *vs*. physician	0.69 (0.38–1.22)	0.202
	Pharmacist *vs*. physician	0.52 (0.37–0.73)	<0.001

Notes: *applies to participants aged 65 years or older; **applies to participants aged 60 years or older; ^a^No nurse had been vaccinated; ‡Odds Ratio (95% confidence interval).

## 4. Discussion

We focused on vaccination coverage of PCPs who are on the frontline of adult immunization and demonstrated suboptimal rates for all examined vaccines. In particular, the observed vaccination coverage against seasonal influenza was 66.4%, for tetanus 62.9% and for pneumococcal pneumonia 70%. Only 12.3% of PCPs aged ≥60 had received a herpes zoster vaccine and only 10% aged ≥65 were fully vaccinated against all four diseases.

Despite the longstanding national and international recommendations and annual campaigns on the importance of vaccinating HCPs against seasonal influenza, the reported vaccine uptake among surveyed PCPs was lower from the minimum recommended target of 75% set by the European Union (EU) [15]. Suboptimal vaccination rates are also observed in recent Greek studies among HCPs in primary care centers (ranged from 40.2% to 56%) [11],[12],[14] and in tertiary-care hospitals (60.2%) [16], as well as in most European countries [17], which reflect the inadequate prevention of the disease transmission in healthcare settings. Only the study by Marinos et al. [18] meets the immunization target (2020–2021 flu season: 76%); however, this may be attributed to the Covid-19 pandemic and its beneficial impact on the intention to vaccinate against influenza [19].

Vaccination against tetanus-diphtheria is recommended for all HCPs in 12 European countries, while in 6 it is mandatory or even a prerequisite for hiring. However, in Greece there are no specific vaccination policies against tetanus-diphtheria (Td) or tetanus-diphtheria-pertussis (Tdap) for HCPs [20]. HCPs lie within the recommendations described in the Greek NAIS for the routine immunization of the general public where periodic boosters in adulthood are required to maintain immunity. We have revealed insufficient, but nonetheless, higher vaccination rates against tetanus compared to previously reported data for Greece (62.9% *vs*. 47.3% for the booster dose with Td [13], and 18% for the Tdap [11]). This may be a result of false self-reported immunization, as has been reported elsewhere, where PCPs inaccurately reported their childhood vaccination against tetanus [11]. However, the higher rate detected in our study may be attributed in large part to the young sample of our study (38.3% aged 19–39 years), where men are more likely to get vaccinated against tetanus during their military service [21]. This suggestion is also consistent with our findings, which show a robust association between younger individuals and greater rates of tetanus vaccination.

Influenza, herpes zoster and pneumococcal infections are considered as the three most important vaccine preventable diseases of aging, causing significant morbidity and-for influenza and pneumococcal disease-mortality in older people [22]. Increasing vaccination coverage of older adults against influenza, herpes zoster and pneumococcal infection has the greatest potential for preventing morbidity and mortality among people aged ≥65 and it can be expected to promote healthy aging [23]. Our findings show that the immunization of older PCPs against influenza are encouraging (70.3% for PCPs ≥50 years old), as they come close to achieving the 75% vaccination coverage target set by EU for older age groups [15]. However, to the best of our knowledge, this is the first study in Greece assessing the vaccination coverage for pneumococcal and zoster vaccines among HCPs, revealing insufficient rates for both vaccines (70.0% for pneumococcal vaccination-far below the target of 90% established by the Healthy People 2020 goals [23], and 12.3% for herpes zoster vaccination-comparable to the vaccination rates of the elderly in Greece (20%) [24], and to those of UK (21.4%) [25]).

The present study has recognized likely determinants of vaccine uptake by PCPs. Being a physician was associated with increased likelihood of influenza or influenza and tetanus vaccination. This finding is consistent with previous studies [26]–[29], where the higher vaccination rates among physicians have been related to their greater confidence in the benefits and safety of vaccines [10]. In terms of sex, men showed significantly higher rates of vaccination against influenza and the combination of influenza and tetanus than women. However, previously published evidence shows inconclusive results regarding the role of sex in vaccine uptake, as higher coverage rates have been observed in both sexes [30].

The Greece's free vaccination policy is a strategy that according to previous research, constitutes an essential component to increase vaccination rates [31]. Furthermore, strategic actions such as promoting campaigns, on-site vaccinations, rewarding programs, declination forms, etc. are implemented in healthcare settings nationwide in order to achieve optimal influenza vaccination rates among HCPs [32]. However, the insufficient rates detected in our study suggest that hesitancy or other determinants, such as socioeconomic status than financial or practical difficulties, may be the prevailing barrier among PCPs [33]. Future studies evaluating the knowledge, attitudes and beliefs of Greek PCPs toward vaccination may help in assessing the extent of vaccine hesitancy among them and add essential information to raise their awareness. Moreover, future studies might also evaluate the possible impact of the experience of PCPs on their vaccination status.

As mentioned earlier, the immunization of HCPs is important for their own protection and for protection of their colleagues and patients. Although relative evidence in Greece is lacking, several studies have shown that unvaccinated HCPs may serve as sources of transmission of vaccine-preventable diseases to patients and colleagues, leading to nosocomial infections and outbreaks [34]. Efforts to strengthen vaccine advocacy and increase coverage will turn the healthcare workforce into an efficient barrier against infectious diseases. In addition, the determination of the PCPs' compliance to self-immunization provides further insight into the extent to which they adhere to guidelines described by the NAIS and might reflect a general communication attitude about vaccines between PCPs and the public [35]. Furthermore, the present study could be a stimulus for the implementation of individualized and targeted interventions to increase PCPs vaccine uptake and promotion with important public health implications. Finally, further research that considers both age and health related criteria would produce valuable knowledge on this topic.

One of the major strengths of the present study is the recruitment of participants from all Greek regions, which is challenging considering the lack of any national electronic database for HCPs and its focus on PCPs. By contrast, major limitations are the convenient sample and the small number of participants aged 60 or over (the right to a pension in Greece can be exercised between 62 and 67 years of age) [36], which might not be representative of the Greek PCPs. Furthermore, the data were self-reported, which may have resulted in social-desirability bias, expressed by overestimation of the vaccination coverage rates.

## 5. Conclusions

Staying up to date with the NAIS recommendations has yet to be fully achieved among PCPs in Greece. Our results provide evidence on PCPs' compliance to self-immunization and underline the need for initiatives to increase the vaccination coverage of PCPs in Greece, which may result in higher vaccination rates of the general population. We suggest that differences in profession and age among PCPs might require tailored approach to increase their vaccination uptake. Targeted educational or training interventions and HCP-focused campaigns might improve PCPs awareness. Finally, our findings might guide the international readership in the necessity of implementing effective vaccination strategies to achieve high vaccination coverage.

## Use of AI tools declaration

The authors declare they have not used Artificial Intelligence (AI) tools in the creation of this article.
